# Dual Mechanical Complications of an Inferior Myocardial Infarction: A Case of Concurrent Left Ventricular Pseudoaneurysm and Ventricular Septal Rupture

**DOI:** 10.7759/cureus.93279

**Published:** 2025-09-26

**Authors:** Houda Mokhlis, Zine-eddine Hamza, Hamid Jalal, Abdessamad Abdou, Abdelmajid Bouzerda, Ali Khatouri

**Affiliations:** 1 Cardiology, Avicenna Military Hospital, Marrakesh, MAR; 2 Cardiovascular Surgery, Avicenna Military Hospital, Marrakesh, MAR

**Keywords:** mechanical complications, myocardial infarction, surgical repair, ventricular free wall rupture, ventricular septal defect (vsd)

## Abstract

We present the case of a 63-year-old male admitted for persistent epigastric pain, initially managed as gastrointestinal in origin. Further evaluation revealed an extensive inferior myocardial infarction, complicated by mechanical complications. Despite intensive medical and surgical management, the patient succumbed to cardiogenic shock postoperatively. This case underscores the importance of early diagnosis and reperfusion in acute coronary syndromes, particularly in atypical presentations. The timing of surgical repair in mechanical complications remains a critical and controversial aspect of management.

## Introduction

Mechanical complications of acute myocardial infarction (MI), such as free wall rupture, ventricular septal defect (VSD), and papillary muscle rupture, are rare but highly fatal events. Their prognosis depends on early recognition, timely intervention, and, most importantly, early reperfusion therapy. Delayed diagnosis - especially when symptoms are atypical - significantly increases the risk of rupture and adverse outcomes. We report a case of inferior MI presenting with atypical symptoms, culminating in fatal mechanical complications [1].

## Case presentation

A 63-year-old man with well-controlled type 2 diabetes and a 25-pack-year smoking history, but no known hypertension or coronary artery disease, presented with severe epigastric pain persisting for four days.

Initial assessment

The patient was conscious and hemodynamically stable at admission (BP 100/75 mmHg and HR 105 bpm). The cardiopulmonary exam was unremarkable, with no murmurs or signs of heart failure.

Investigations

**Figure 1:**
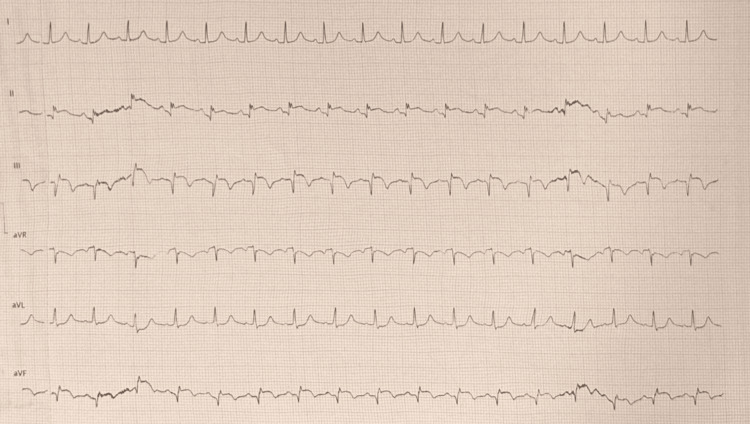
Electrocardiogram recorded at admission, demonstrating ST-segment elevation in leads II, III, and aVF, consistent with acute inferior myocardial infarction

**Figure 2:**
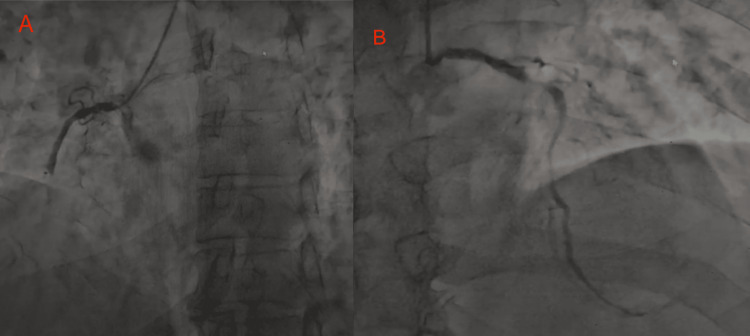
Coronary angiography (A) Total occlusion of the right coronary artery (RCA); (B) Significant stenotic lesion of the left anterior descending artery (LAD).

Electrocardiogram (EKG) showed changes consistent with an evolving inferior MI (Figure 1). Transthoracic echocardiogram (TTE) demonstrated segmental wall motion abnormalities in the inferior and inferolateral walls, with preserved biventricular size and function. Coronary angiography revealed thrombotic occlusion of the right coronary artery (RCA), associated with a lesion of the ostial left anterior descending artery (LAD), as well as sequential plaques in the mid-LAD (Figure 2). The circumflex artery was small in size and exhibited diffuse lesions.

During the procedure, the patient developed hemodynamic instability, requiring supportive measures. It was therefore decided to initiate tirofiban for 12 hours and to defer the intervention. Dual antiplatelet therapy, therapeutic-dose low molecular weight heparin, and a statin were started.

Clinical course

The patient developed new-onset atrial fibrillation, which reverted to sinus rhythm after amiodarone. On day 2, the patient presented with septic shock of undetermined origin, with an elevated procalcitonin level six times above normal, associated with acute kidney injury (estimated glomerular filtration rate (eGFR): 11/65 mL/min) and signs of bilateral lower limb hypoperfusion, requiring the initiation of vasoactive support (norepinephrine) and empiric antibiotic therapy (imipenem). Procalcitonin later dropped to 0.4 ng/mL; eGFR improved to 55 mL/min, although a systemic inflammatory response to acute MI could not be entirely excluded. The patient also experienced episodes of flash pulmonary edema (FPE), accompanied by the appearance of a holosystolic regurgitant murmur radiating in a circular pattern.

Echocardiographic findings

**Figure 3:**
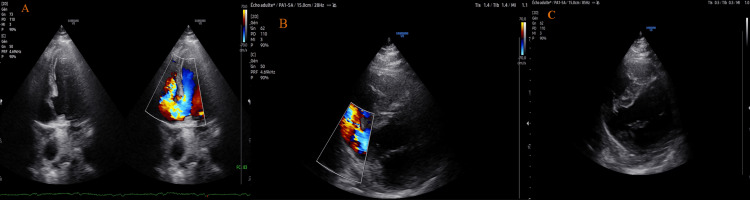
Transthoracic echocardiogram (A) Apical four-chamber view with color Doppler demonstrating ventricular septal rupture; (B) Parasternal short-axis view with color Doppler showing left-to-right shunt across the rupture; (C) Parasternal short-axis view revealing inferior wall rupture contained by the pericardium.

**Figure 4:**
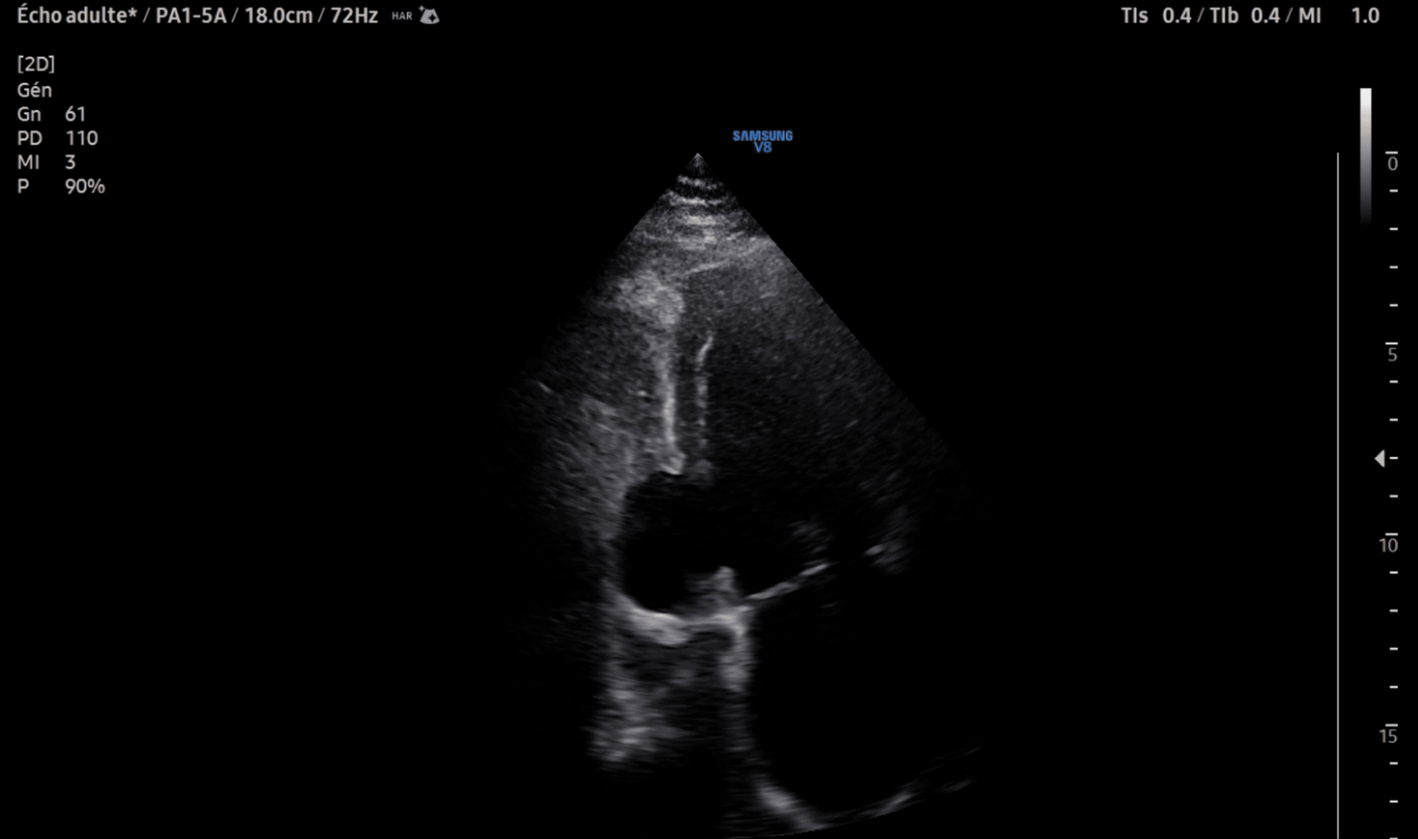
Two-chamber transthoracic echocardiographic view showing a left ventricular pseudoaneurysm consistent with contained myocardial rupture

In light of these findings, the follow-up echocardiographic examination revealed a large posterior VSD shunting from left to right, visible in the four-chamber view as well as in the parasternal short-axis view (Figure 3). Loss of continuity in the inferior wall was also seen (Figure 4), creating the appearance of a false or pseudoaneurysm resulting from a contained rupture, unlike a true aneurysm, which is a well-demarcated, transmural scar with thinned, dyskinetic myocardium and a wide neck, often affecting overall myocardial performance.

Management and outcome

After the multidisciplinary team decision and hemodynamic stabilization, the patient underwent surgery on day 11 after diagnosis. The main challenge was the localization of the VSD and the posterior rupture, which required cardiac displacement to achieve better exposure. This maneuver allowed visualization of the left ventricular rupture, which was sealed by a blood clot.

**Figure 5:**
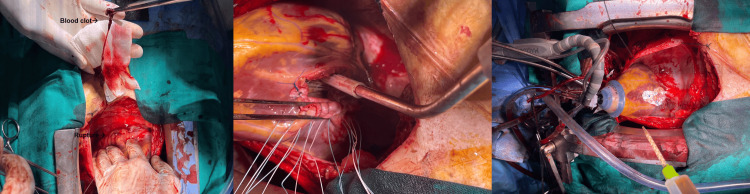
Intraoperative photographs showing surgical repair of both ventricular septal defect and pseudoaneurysm

They then resected the entire infarcted wall and identified a large posterior VSD. Subsequently, the defect was closed using a biological patch. The ruptured myocardial wall was repaired with the same patch, followed by approximation of the edges and reinforcement with biological adhesive (Figure 5). Finally, a single coronary artery bypass graft (CABG) of the LAD, using the left internal mammary artery (LIMA), was performed. Weaning from cardiopulmonary bypass was uneventful, under low doses of norepinephrine and dobutamine.

However, despite mechanical support, including inotropes and intra-aortic balloon pump (IABP), the patient developed cardiogenic shock and died within 10 hours postoperatively.

## Discussion

Mechanical complications following MI are rare (1%-3%) but carry high mortality if not promptly managed. Risk factors for myocardial rupture include older age, female sex, first MI, anterior infarct (though this case involved an inferior MI), absence of collateral circulation, persistent ST-segment elevation, and right ventricular involvement [2].

The myocardial tear is exacerbated by shear stress and the hypercontractile force of the myocardium adjacent to the infarcted zone. Free wall rupture is further promoted by fibrinolytic therapy, which tends to favor hemorrhagic transformation and dissection rather than coagulative necrosis, as well as by the use of nonsteroidal anti-inflammatory drugs (NSAIDs) [3] and early physical exertion. In our case, the patient did not receive fibrinolytic therapy or NSAIDs, thereby excluding these factors as contributors.

A 2005 study in the American Journal of Cardiology identified persistent ST-segment elevation beyond 72 hours and right ventricular involvement as risk factors for ventricular septal rupture [4]. Conversely, diabetes and prior MI were negative predictors. Ischemic preconditioning in these patients was proposed as a possible explanation for these findings.

Once the diagnosis of cardiac rupture is confirmed, the main challenge lies in determining the optimal timing for surgical repair. Early surgical intervention is often necessary due to the high risk of hemodynamic deterioration and death; however, operating during the acute phase - typically within the first few days - can be technically challenging due to the friability of the necrotic tissue, which increases the risk of suture dehiscence and residual defects. Conversely, delayed surgery, when feasible, allows for some degree of tissue healing and fibrotic reinforcement, potentially improving surgical outcomes. The decision must therefore be individualized, balancing the urgency of intervention with the patient’s hemodynamic status, the extent of the rupture, and the risk of further deterioration [5].

Recent guidelines from the American Heart Association (AHA) and the European Society of Cardiology (ESC) highlight the lack of consensus regarding the optimal timing for surgical intervention [6-8]. While they acknowledge that early surgical repair is associated with higher mortality rates, a delayed approach may offer improved outcomes in selected patients. However, in hemodynamically unstable patients, medical management alone may compromise survival by delaying surgery - particularly in cases of refractory cardiogenic shock, where urgent intervention becomes critical.

Regarding post-infarction VSDs, the 2023 European guidelines on acute coronary syndromes recommend early surgery for patients presenting with refractory cardiogenic shock or persistent right ventricular dysfunction [9]. In other patients, a delayed approach is advised - preferably beyond the seventh day after diagnosis - and, if needed, preceded by mechanical circulatory support (MCS). Delaying repair facilitates tissue healing and patch suturing. In contrast, for pseudoaneurysms, a shorter delay before surgical repair is warranted, even in hemodynamically stable patients, due to their unpredictable course and high risk of complete rupture.

Surgical timing must balance between the risks of organ failure and/or sudden hemodynamic deterioration and the potential benefit of allowing better tissue healing, which may lead to improved surgical outcomes.

A recent update published in the Journal of the American College of Cardiology (JACC) on mechanical complications of acute coronary syndromes emphasizes the emerging role of temporary MCS before surgery, but notes limited evidence of benefit [10,11]. Percutaneous defect closure is also an emerging alternative for suitable anatomy, high-risk patients, or as a bridging strategy to delay surgical intervention.

IABP support remains first-line mechanical circulatory assistance in patients with post-infarction VSDs, reducing left-to-right shunting by decreasing left ventricular afterload, preserving cardiac output, and maintaining systemic perfusion [12].

In cases of free wall rupture with circulatory collapse, veno-arterial extracorporeal membrane oxygenation (VA-ECMO), or other forms of MCS, may allow hemodynamic stabilization as a bridge to definitive surgical repair. For stable patients with a contained rupture, close monitoring and strict blood pressure control are essential; percutaneous closure should be considered as a potential alternative if inoperable [11].

Once the indication for surgical repair has been established, another dilemma arises: whether to perform concomitant myocardial revascularization - a topic that has long been a subject of debate. A 2022 meta-analysis published in the Annals of Cardiothoracic Surgery, which included over 4,312 patients with mechanical complications following acute coronary syndromes, found that 49% underwent concomitant CABG [13]. The analysis found little to no benefit of concomitant CABG on early and late postoperative mortality.

In another study published in the Journal of Cardiac Surgery in 2020 [14], a higher mortality rate was observed in patients undergoing concomitant CABG during ischemic VSD repair, although this difference was not statistically significant during long-term follow-up.

Conversely, other studies, including this study published in the European Journal of Cardio-Thoracic Surgery [15], suggest that concomitant revascularization of non-culprit vessels in multivessel disease patients improves survival by protecting the residual myocardium.

The debate remains unresolved; what is clear, however, is that a balance must be struck between the benefits of revascularization and the risks associated with prolonged cardiopulmonary bypass. 

Despite mixed evidence regarding CABG in mechanical complications of MI, our surgical team performed single-vessel revascularization of the LAD alongside VSD and pseudoaneurysm repair. This approach aimed to protect residual myocardium while minimizing operative time and complexity, balancing surgical risk with potential benefit.

In this patient, delayed recognition of MI due to atypical presentation likely led to the catastrophic complication. The rupture involved the inferoposterior wall of the left ventricle, resulting in a large posterior VSD - a rare but fatal event.

Timing of surgical repair

There is no consensus on the optimal timing for surgical repair in post-infarction VSD. While urgent surgery is often required, outcomes may be improved by temporary stabilization, in selected patients, using MCS.

## Conclusions

This case underscores the devastating impact of delayed recognition of MI, particularly when initial symptoms are atypical. Mechanical complications, such as free wall rupture and post-infarction VSD, carry a high risk of mortality and require prompt echocardiographic evaluation and coordinated surgical planning. Early and effective reperfusion remains the most reliable strategy for preventing these complications and improving clinical outcomes.
